# Effect of Obesity and High-Density Lipoprotein Concentration on the Pathological Characteristics of Alzheimer’s Disease in High-Fat Diet-Fed Mice

**DOI:** 10.3390/ijms232012296

**Published:** 2022-10-14

**Authors:** Moonseok Choi, Dongsoo Kim, Young-Jin Youn, Junghwa Ryu, Yun Ha Jeong

**Affiliations:** 1 Department of Neurodegenerative Diseases Research Group, Korea Brain Research Institute, 61, Cheomdan ro, Dong gu, Daegu 41062, Korea; 2 Department of Radiology, School of Medicine, Catholic University of Daegu, 33, Duryugongwon-ro 17-gil, Nam-gu, Daegu 42472, Korea

**Keywords:** Alzheimer’s disease, high-fat diet, obesity, high-density lipoprotein, apolipoprotein AI, amyloid plaque

## Abstract

The typical pathological features of Alzheimer’s disease (AD) are the accumulation of amyloid plaques in the brain and reactivity of glial cells such as astrocytes and microglia. Clinically, the development of AD and obesity are known to be correlated. In this study, we analyzed the changes in AD pathological characteristics in 5XFAD mice after obesity induction through a high-fat diet (HFD). Surprisingly, high-density lipoprotein and apolipoprotein AI (APOA-I) serum levels were increased without low-density lipoprotein alteration in both HFD groups. The reactivity of astrocytes and microglia in the dentate gyrus of the hippocampus and fornix of the hypothalamus in 5XFAD mice was decreased in the transgenic (TG)-HFD high group. Finally, the accumulation of amyloid plaques in the dentate gyrus region of the hippocampus was also significantly decreased in the TG-HFD high group. These results suggest that increased high-density lipoprotein level, especially with increased APOA-I serum level, alleviates the pathological features of AD and could be a new potential therapeutic strategy for AD treatment.

## 1. Introduction

Alzheimer’s disease (AD) is a typical neurodegenerative disease and the most common form of dementia. The characteristic pathological features of AD include amyloid plaques in the brain, hyperphosphorylation of tau tangles in neurons, reactivation of glial cells (such as astrocytes and microglia), cognitive dysfunction, and behavioral disturbances [1,2]. According to various reports, the number of patients with AD is expected to increase to around 150 million by 2050; around one-third of the global population over the age of 65 will develop AD [3]. Furthermore, the incidence rate of AD is approximately three times higher in women than in men [4,5].

In recent clinical studies, a high body mass index (BMI) among specific populations was associated with a preventive role in AD [6]. The underweight population, with BMI < 20 (unit, kg/m^2^), had a higher risk of AD than the healthy weight population, while the overweight population, with BMI ≥ 40, had a lower risk of AD [7]. Several reviews and meta-analyses have reported a negative correlation between the risk of AD mortality and progression and BMI [8,9,10]. Compared with a healthy weight population, the underweight population, with BMI < 20, had an approximately 50% higher risk of AD mortality, while the overweight population, with BMI ≥ 40, had an approximately 10% lower risk of AD mortality [7,11]. However, the relationships and mechanisms between BMI and the risk of AD progression and mortality have not been fully elucidated.

Several studies have investigated the effect of obesity on AD pathological characteristics using a high-fat diet (HFD) in various AD mouse models, such as APP/PS1, 3xTg-AD, and 5XFAD [12,13,14]. These studies showed that a HFD affected AD pathological characteristics differently according to the starting age of HFD intake, period of HFD intake, and sex. HFD feeding before 3 months of age had protective effects on amyloid plaque deposition and cognitive decline [13,14]. In contrast, other studies revealed that HFD-fed mice exhibited enhanced accumulation of β-amyloid (Aβ), reactivation of glial cells, and cognitive impairment [12]. However, the mechanism of HFD consumption on the pathological characteristics of AD is not yet fully understood.

In this study, we investigated the effect of HFD-induced obesity on the pathological characteristics of AD by analyzing the physiological changes in metabolic function and adipogenesis, as well as the pathological changes in the brain, in 5XFAD mice.

## 2. Results

**Figure 1:**
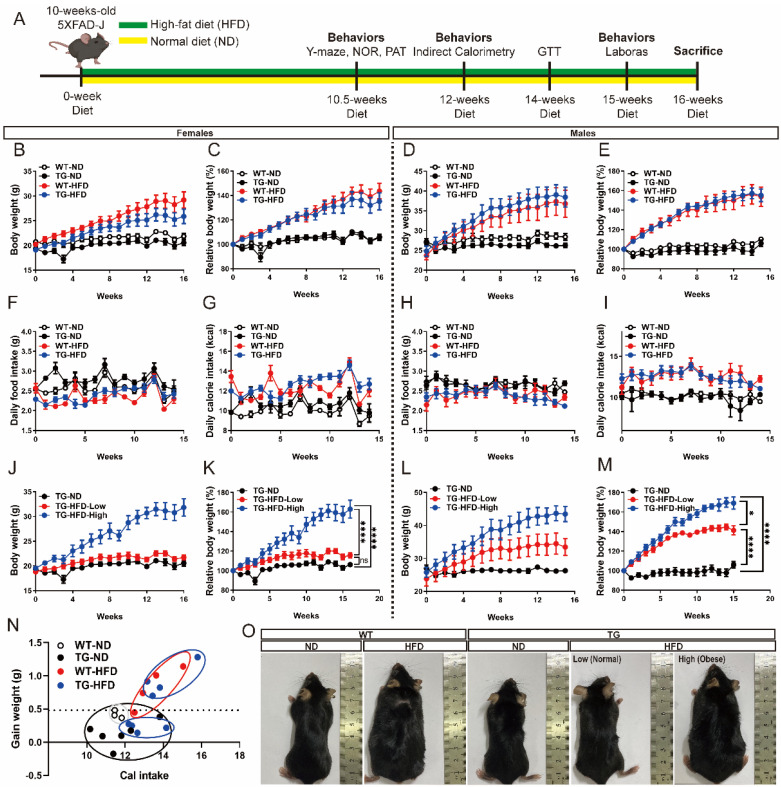
Body weight changes under a high-fat diet (HFD) in the 5XFAD Alzheimer’s disease transgenic mouse model. Experimental scheme (**A**). Weekly measurements of the body weight (**B**), change in body weight (**C**), daily food intake (**D**), and daily calorie intake (**E**) in female mice. Weekly measurements of the body weight (**F**), change in body weight (**G**), daily food intake (**H**), and daily calorie intake (**I**) in male mice. Body weight of the female mice in divided HFD-fed groups (**J**) and change in body weight (**K**). Body weight of the male mice in divided HFD-fed groups (**L**) and change in body weight (**M**). Calorie efficiency chart for weight gain in female mice (**N**). The photographs represent the body shape of the female mice in each group (**O**). ns (not significant), * *p* < 0.05, and **** *p* < 0.0001 (one-way analysis of variance with Tukey post hoc analysis). Data are presented as the mean ± standard error of the mean. The number of mice in each group ranged 7–12 for females and 4–8 for males. ND, normal chow; WT, wildtype; TG transgenic.

To determine the effect of obesity on AD progression, a diet containing 60% fat (HFD) or 10% normal chow (ND) was fed to male and female 5XFAD AD model transgenic (TG) mice and age-matched wild-type (WT) mice from 10 to 26 weeks of age. At 10.5 weeks after diet initiation, Y-maze, novel object recognition, and passive avoidance tests were performed to analyze changes in cognitive function. At 12 weeks after diet initiation, indirect calorimetry (IC) was performed to continuously analyze the changes in energy expenditure (EE). At 14 weeks after diet initiation, the glucose tolerance test (GTT) was performed to measure the body’s response to sugar. At 15 weeks after diet imitation, the Laboratory Animal Behavior Observation, Registration, and Analysis System (LABORAS) test was performed to analyze changes in various locomotor behaviors. At 16 weeks after diet imitation, all mice groups were sacrificed for serum cholesterol analysis. The experimental scheme and timeline are summarized in Figure 1A.

### 2.1. Body Weight Changes under a High-Fat Diet in 5XFAD Mice

During the diet period, the body weight and food intake of each mouse was measured on a weekly basis. The HFD group showed a greater increase in body weight than the ND group. In female WT mice, body weight was significantly greater in the WT-HFD group than in the WT-ND group diet initiation. Similarly, among female TG mice, body weight was significantly greater in the TG-HFD group than in the TG-ND group (Figure 1B,C). Among male WT mice, body weight was significantly greater in the WT-HFD group than in the WT-ND group after diet initiation. Similarly, among male TG mice, body weight was significantly greater in the TG-HFD group than in the TG-ND group (Figure 1D,E). No significant difference was found in food intake between male and female mice, whereas calorie intake was higher in HFD groups than in ND groups (Figure 1F–I). The HFD groups were further divided into TG-HFD-Low and TG-HFD-High groups according to body weight change. Interestingly, among female mice, compared to that in the TG-ND group, the TG-HFD-Low group showed no difference in body weight (i.e., normal weight) while the TG-HFD-High group showed significantly increased body weight compared to that in the TG-ND and TG-HFD-Low groups (i.e., obesity) (Figure 1J,K). In contrast, in male TG mice, the body weight was significantly increased in both the TG-HFD-Low and TG-HFD-High groups compared to that in the TD-ND group (Figure 1L,M). The calorie efficiency chart of the female TG-HFD mice showed a significant separation into two groups when compared to that in the other groups (Figure 1N). Representative images of each group are shown in Figure 1O. These results revealed that although obesity was generally induced by feeding a HFD, the TG-HFD mice, especially female mice, could be divided into two groups (obese and normal) based on body weight changes. As this distinction was clearer in the female mice than in the male mice, and for additional reasons covered in the discussion, the female mice were analyzed as the main experimental group.

### 2.2. Metabolic Changes under the High-Fat Diet in 5XFAD Mice

**Figure 2:**
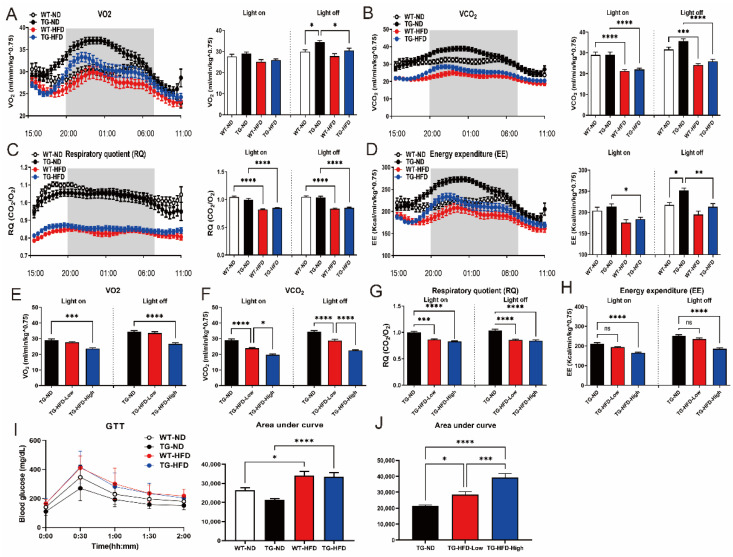
Analysis of the metabolic changes under a high-fat diet (HFD) by indirect calorimetry and the glucose tolerance test (GTT) in the 5XFAD Alzheimer’s disease transgenic mouse model. Analysis of oxygen consumption (vO2) normalized to the body weight in female mice, plotted at 30 min intervals during 1 day (**A**, left panel), and vO2 in light-on and -off conditions (**A**, right panel). Carbon dioxide production (vCO2) plotted at 30 min intervals during 1 day (**B**, left panel) and vCO2 in light-on and -off conditions (**B**, right panel). Respiratory quotient (RQ) plotted at 30 min intervals during 1 day (**C**, left panel) and RQ in light-on and -off conditions (**C**, right panel). Energy expenditure (EE) plotted at 30 min intervals during 1 day (**D**, left panel) and EE in light-on and -off conditions (**D**, right panel). Analysis of female mice in the divided HFD-fed groups (TG-HFD-High and -Low): vO2 in light-on and -off conditions (**E**), vCO2 in light-on and -off conditions (**F**), RQ in light-on and -off conditions (**G**), and EE in light-on and -off conditions (**H**). Blood glucose levels in female mice (**I**, left panel), with an analysis of the area under curve (AUC) in the TD and HFD groups (**I**, right panel) and in the divided HFD-fed groups (**J**). ns (not significant), * *p* < 0.05, ** *p* < 0.01, *** *p* <0.001, and **** *p* < 0.0001 (one-way analysis of variance with Tukey post hoc analysis). Data are presented as the mean ± standard error of the mean. The number of mice in each group ranged 7–12. ND, normal chow; WT, wildtype; TG transgenic.

Since the TG-HFD group could be divided into two groups, TG-HFD-High and TG-HFD-Low, based on body weight changes, we attempted to confirm whether the metabolic processes also differed between these two groups in analyses of IC and GTT. According to IC results, oxygen consumption (vO_2_) was significantly higher in the TG-ND group than in the WT-ND group during the light-off period. Surprisingly, vO_2_ was restored in the TG-HFD group. However, no significant differences were found between any of the groups during the light-on period (Figure 2A). Carbon dioxide production (vCO_2_) was significantly lower in HFD groups than in ND groups during light-on and -off periods. Therefore, vCO_2_ was only influenced by a HFD and not by genetic background (Figure 2B). The respiratory quotient (RQ) was significantly lower in HFD groups than in ND groups during both light-on and -off periods. Thus, the RQ was also only influenced by a HFD and not by genetic background, as observed for vCO_2_ (Figure 2C). EE was significantly higher in the TG-ND group than that in the WT-ND group during the light-off period. Similar to that for vO_2_, EE was restored in the TG-HFD group (Figure 2D). Additionally, vO2 was significantly lower in the TG-HFD-High group than in the TG-ND group during light-on and -off periods (Figure 2E). However, vO2 did not differ between TG-ND and TG-HFD-Low groups. vCO2 was significantly lower in the TG-HFD-High group than in the TG-ND and TG-HFD-Low groups during light-on and -off periods (Figure 2F). The RQ was significantly lower in both the TG-HFD-Low and TG-HFD-High groups than in the TG-ND group, but there were no differences between the TG-HFD-Low and TG-HFD-High groups, during light-on and light-off periods (Figure 2G). EE was significantly lower in the TG-HFD-High group than in the TG-ND group during light-on and -off periods (Figure 2H), but there were no differences between the TG-ND and TG-HFD-Low groups. According to the GTT analysis, the blood glucose level was significantly higher in HFD groups than in ND groups (Figure 2I). Surprisingly, a significant difference in the blood glucose level was detected between the TG-HFD-High and TG-HFD-Low groups (Figure 2J). These results showed that energy metabolism was increased in 5XFAD AD model mice, and it was restored through a HFD, despite the presence of glucose metabolism disturbance due to obesity induction. These phenomena were more pronounced in the TG-HFD-High group.

### 2.3. Fat Pathology and Blood Cholesterol Changes under a High-Fat Diet in 5XFAD Mice

**Figure 3:**
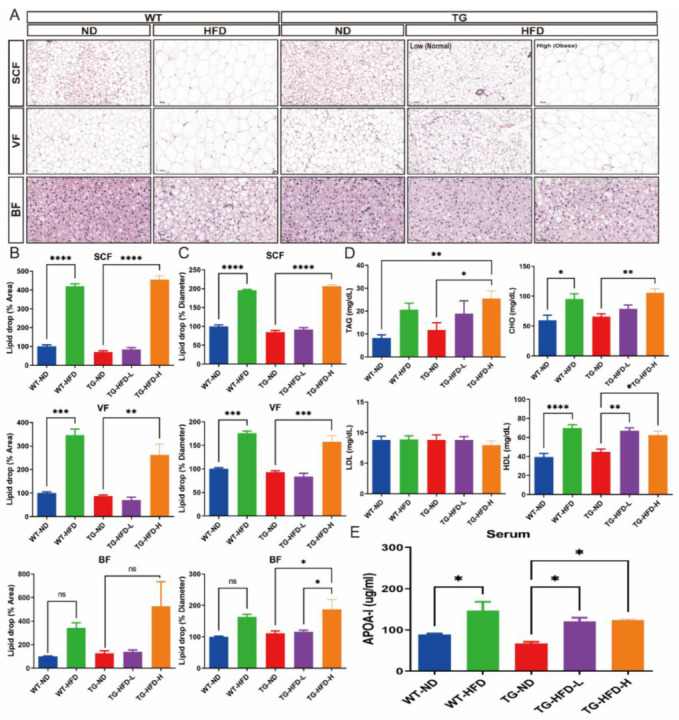
Analysis of fat pathology and blood cholesterol changes under a high-fat diet (HFD) in the 5XFAD Alzheimer’s disease transgenic mouse model. Represent images of subcutaneous fat (SCF), visceral fat (VF), and brown fat (BF) in female mice (**A**); quantitative estimation of the mean percent area of the lipid droplets in SCF (**B**, top panel), VF (**B**, middle panel), and BF (**B**, bottom panel); and quantitative estimation of the mean diameter area of the lipid droplets in SCF (**C**, top panel), VF (**C**, middle panel), and BF (**C**, bottom panel). Blood analysis of triacylglycerol, cholesterol, low-density lipoprotein, and high-density lipoprotein (**D**). Enzyme-linked immunoassay analysis of the serum levels of apolipoprotein AI (APOA-I) (**E**). ns (not significant), * *p* < 0.05, ** *p* < 0.01, *** *p* < 0.001, and **** *p* < 0.0001 (one-way analysis of variance with Tukey post hoc analysis). Data are presented as the mean ± standard error of the mean. The number of mice in each group was 3 for the fat tissue analyses, 6–7 for the blood analyses, and 4 for the APOA-I measurement. ND, normal chow; WT, wildtype; TG transgenic.

To analyze changes in adipose tissue characteristics after obesity induction, the size of the lipid droplets of subcutaneous fat (SCF), visceral fat (VF), and brown fat (BF) was analyzed using hematoxylin and eosin (H&E) staining. Representative images of each adipose tissue sample are shown in Figure 3A. The percent area of the lipid droplets of SCF and VF was significantly greater in the WT-HFD group than in the WT-ND group and in the TG-HFD-High group, but not the TG-HFD-Low group, than in the TG-ND group. The percent area of the lipid droplets of BF was also increased by the HFD, but the difference failed to reach significance (Figure 3B). The percent diameters of the lipid droplets of SCF and VF were significantly greater in the WT-HFD group than in the WT-ND group. Furthermore, the percent diameters of the lipid droplets of SCF, VF, and BF were significantly greater in the TG-HFD-High group than in the TG-ND group, while there were no differences between TG-HFD-Low and TG-ND groups (Figure 3C). These results indicate that although obesity was induced by HFD, the size of the lipid droplets differed between the TG-HFD-High and TG-HFD-Low groups, which were divided based on the body weight change.

In addition, changes in lipid composition, including triacylglycerol (TAG), total cholesterol (CHO), low-density lipoprotein (LDL) cholesterol, and high-density lipoprotein (HDL) cholesterol, in the blood were analyzed. The serum level of TAG was significantly higher in the TG-HFD-High group than in the WT-ND and TG-ND groups. However, the serum TAG level in the WT-HFD and TG-HFD-Low groups was not significantly different from that in the WT-ND group (Figure 3D). The serum CHO level was significantly higher in the WT-HFD group than in the WT-ND group. Among TG groups, the serum CHO level was significantly higher in the TG-HFD-High group than in the TG-ND group (Figure 3D). However, the serum LDL levels in the WT-HFD, TG-ND, TG-HFD-Low, and TG-HFD-High groups did not significantly differ from those in the WT-ND group. Surprisingly, the serum HDL level was significantly higher in the WT-HFD group than in the WT-ND group. Among TG groups, the serum HDL level was significantly higher in the TG-HFD-Low and TG-HFD-High groups than in the TG-ND group (Figure 3D). In addition, the serum apolipoprotein AI (APOA-I) level was significantly higher in the WT-HFD group than in the WT-ND group. APOA-I is the major apolipoprotein of HDL in periphery. Among TG groups, the serum APOA-I level was significantly higher in the TG-HFD-Low and TG-HFD-High groups than in the TG-ND group (Figure 3E), similar to that for the serum HDL level. These results suggest that HFD feeding induces an increase in HDL and APOA-I levels, but not the LDL level, in the serum of 5XFAD AD model mice.

### 2.4. Reactivity of Astrocytes and Microglia in the Hippocampal Dentate Gyrus and Hypothalamus under a High-Fat Diet in 5XFAD Mice

**Figure 4:**
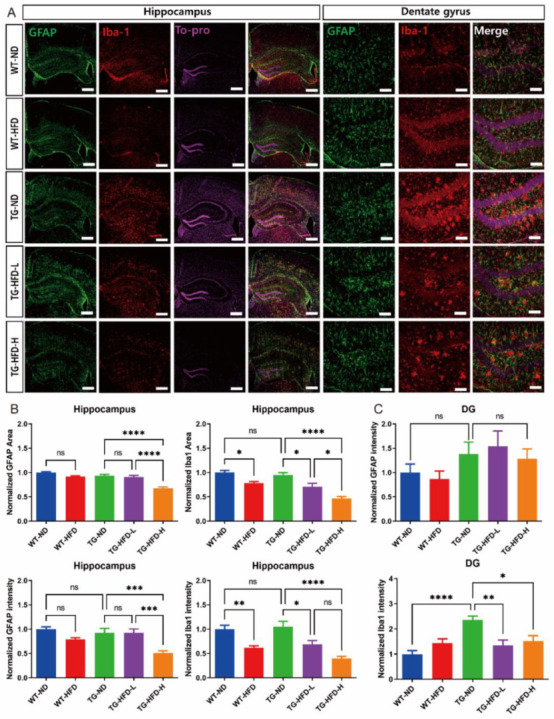
Analysis of astrocyte and microglia reactivity in the hippocampal dentate gyrus (DG) under a high-fat diet (HFD). Representative immunofluorescence images of glial fibrillary acidic protein (GFAP) and ionized calcium binding adaptor molecule 1 (Iba1) staining in the hippocampus of female mice. Scale bars = 100 μm (**A**). Analysis of the stained GFAP area and its intensity in the entire hippocampus (**B**, left panel). Analysis of the stained Iba1 area and its intensity in the entire hippocampus (**B**, right panel). Analysis of the stained GFAP (**C**, top panel) and Iba1 (**C**), bottom panel) intensities in the hippocampal DG. ns (not significant), * *p* < 0.05, ** *p* < 0.01, *** *p* < 0.001, **** *p* < 0.0001 (one-way analysis of variance with Tukey post hoc analysis). Data are presented as the mean ± standard error of the mean. The number of mice in each group ranged 9–10. ND, normal chow; WT, wildtype; TG transgenic.

To determine the effect of HFD-associated increases in serum HDL and APOA-I levels on the pathological characteristics of AD such as the reactivity of astrocytes and microglia in the hippocampus and hypothalamus, we performed immunofluorescence staining with glial fibrillary acidic protein (GFAP), an astrocyte-specific marker protein, and ionized calcium binding adaptor molecule 1 (Iba1), a microglia-specific marker protein. Representative images of stained brain tissues in the hippocampus are shown in Figure 4A. In the entire hippocampal area, the normalized area and intensity of GFAP expression were not different between TG-ND and WT-ND groups; however, they were significantly lower in the TG-HFD-High group than in the TG-ND group. The normalized area and intensity of Iba1 expression were not different between TG-ND and WT-ND groups. In the HFD-fed groups, the normalized area and intensity of Iba1 expression were significantly lower in the WT-HFD group than in the WT-ND group. Similarly, the normalized area and intensity of Iba1 expression were lower in the TG-HFD-High group than in the TG-ND group. Moreover, the normalized area and intensity of GFAP expression and the normalized area of Iba1 expression were significantly lower in the TG-HFD-High group than in the TG-HFD-Low group (Figure 4B). In the hippocampal dentate gyrus (DG) area, the normalized intensity of GFAP expression did not show any changes. However, the normalized intensity of Iba1 expression was significantly higher in the TG-ND group than in the WT-ND group. Interestingly, Iba1 expression was significantly lower in both the TG-HFD-Low and TG-HFD-High groups than in the TG-ND group (Figure 4C). These results suggest that the reactivity of glial cells was higher in the hippocampal DG region of 5XFAD mice, although it was restored by HFD feeding, especially in the microglia.

**Figure 5:**
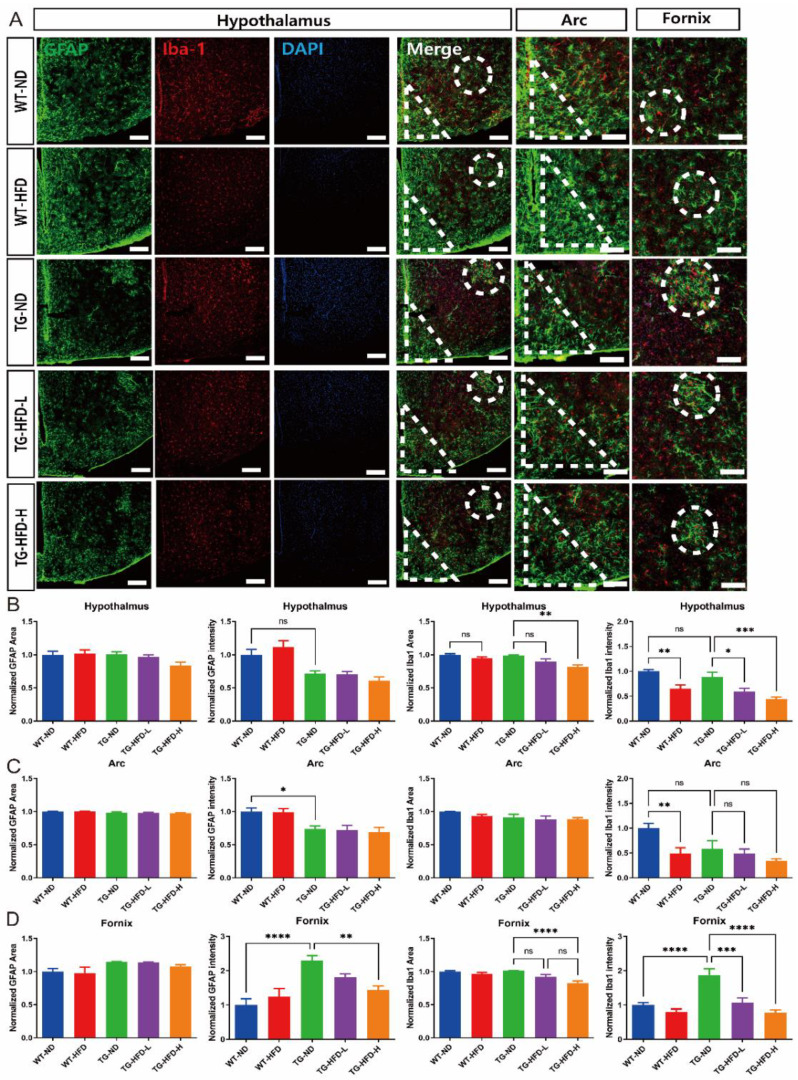
Analysis of astrocyte and microglia reactivity in the hypothalamus under a high-fat diet (HFD). Representative immunofluorescence images of glial fibrillary acidic protein (GFAP) and ionized calcium binding adaptor molecule 1 (Iba1) staining in the hypothalamus of female mice. Dotted circles indicate the fornix region and triangles indicate the arc region. Scale bars = 100 μm (**A**). Analysis of the stained GFAP area and its intensity in the entire hypothalamus (**B**, left panel). Analysis of the stained Iba1 area and its intensity in the entire hypothalamus (**B**, right panel). Analysis of the stained GFAP area and its intensity in the hypothalamic arcuate nucleus (arc) region (**C**, left panel). Analysis of the stained Iba1 area and its intensity in the arc region (**C**, right panel). Analysis of stained the GFAP area and its intensity in the fornix region (**D**, left panel). Analysis of the stained Iba1 area and its intensity in the fornix region (**D**, right panel). ns (not significant), * *p* < 0.05, ** *p* < 0.01, *** *p* < 0.001, and **** *p* <0.0001 (one-way analysis of variance with Tukey post hoc analysis). Data are presented as the mean ± standard error of the mean. The number of mice in each group ranged 8–10. ND, normal chow; WT, wildtype; TG transgenic.

To elucidate the reasons for the weight grouping after HFD initiation in the 5XFAD mice, immunofluorescence staining was performed in the hypothalamus to observe glial cell abnormalities. Representative images of stained brain tissue in the hypothalamus are shown in Figure 5A. The normalized area and intensity of GFAP expression in the entire hypothalamic area did not show changes in any of the HFD groups compared to that in the WT-ND group. However, the normalized intensity of Iba1 expression was significantly lower in the WT-HFD group than in the WT-ND group. Furthermore, the normalized intensity of Iba1 expression was significantly lower in both the TG-HFD-Low and TG-HFD-High groups than in the TG-ND group (Figure 5B). In the hypothalamic arcuate nucleus (arc) region, the normalized area of GFAP and Iba1 expression did not show changes in any of the HFD groups compared to that in the WT-ND group. The normalized intensity of GFAP expression was significantly lower in the TG-ND group than in the WT-ND group. The normalized intensity of Iba1 expression was significantly lower in the WT-HFD group than in the WT-ND group. However, the normalized intensity of Iba1 expression did not show any differences between TG groups (Figure 5C). In the hypothalamic fornix region, the normalized area of GFAP expression did not exhibit changes in any of the HFD groups compared to that in the WT-ND group. However, the normalized intensity of GFAP expression was significantly higher in the TG-ND group than in the WT-ND group. Moreover, the normalized intensity of GFAP expression was significantly lower in the TG-HFD-High group than in the TG-ND group. The normalized area of Iba1 expression was significantly lower only in the TG-HFD-High group than in the TG-ND group in the hypothalamic fornix region. Interestingly, the normalized intensity of Iba1 expression was significantly higher in the TG-ND group than in the WT-ND group, whereas it was prominently lower in both the TG-HFD-Low and TG-HFD-High groups (Figure 5D). These results indicate that the reactivity of glial cells (a pathological characteristic of AD) was restored in the hippocampal DG and hypothalamic fornix regions after HFD initiation.

### 2.5. Changes in Behavior and Amyloid Plaque Number under a High-Fat Diet in 5XFAD Mice

**Figure 6:**
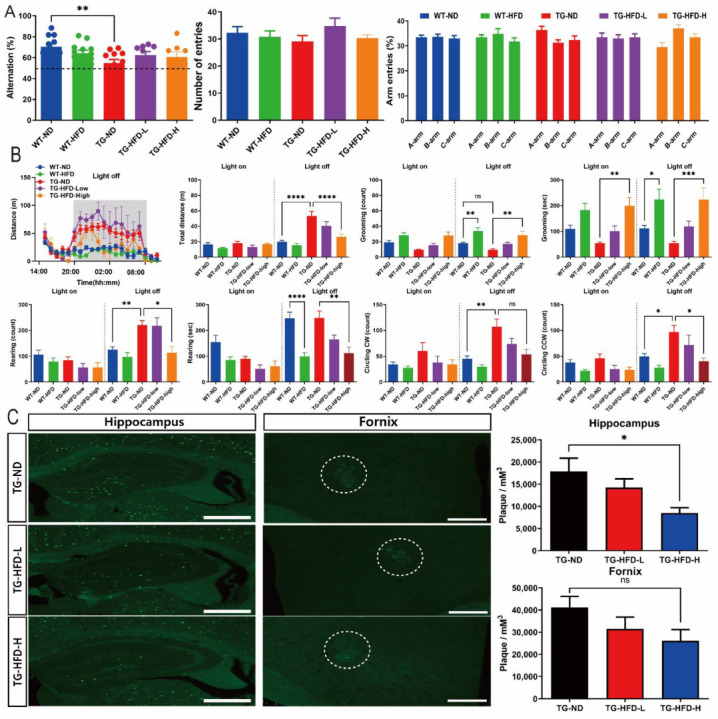
Investigation of behavioral changes and amyloid plaque number under a high-fat diet (HFD). Bar graphs of spontaneous alternations in the Y-maze (**A**, left panel), number of entries (**A**, center panel), and percent of arm entries (**A**, right panel). Analysis of the general behavior of mice using the Laboratory Animal Behavior Observation, Registration and Analysis System (LABORAS) test. Total distance, duration, and frequency of grooming, rearing, and clockwise and counterclockwise circling in light-on and -off conditions (**B**). Representative images of thioflavin-S staining in the hippocampal and fornix regions (**C**, left panel), bar graph of the amyloid plaque density in the hippocampal and fornix regions (**C**, right panel). ns (not significant), * *p* < 0.05, ** *p* < 0.01, *** *p* < 0.001, and **** *p* < 0.0001 (one-way analysis of variance with Tukey post hoc analysis). Data are presented as the mean ± standard error of the mean. The number of mice in each group was 8–12 for the Y-maze, 6–12 for the LABORAS test, and 5 for thioflavin-S staining. ND, normal chow; WT, wildtype; TG transgenic.

To determine the effect of HFD on the behavioral characteristics of AD, such as working memory and basic mouse behavior, we performed Y-maze and LABORAS tests. In the Y-maze test, the percent of spontaneous alternations was significantly lower in the TG-ND group than in the WT-ND group, without any change in the total number of entries and percent of each arm entry. However, there was no effect of HFD on the percent of spontaneous alternations, for both TG-HFD-Low and TG-HFD-High groups, when compared to that in the TG-ND group (Figure 6A). In the LABORAS test, the total distance was significantly greater in the TG-ND group than in the WT-ND group during the light-off period (8:00 p.m. to 08:00 a.m.). This increase in total distance in the TG-ND group was restored in the TG-HFD-High group. The number of grooming episodes during the light-off period was significantly higher in the WT-HFD group than in the WT-ND group and in the TG-HFD-High group than in the TG-ND group. Similarly, the latency of grooming during the light-off period was markedly longer in the WT-HFD group than in the WT-ND group and in the TG-HFD-High group than in the TG-ND group. The number of rearing episodes was significantly higher in the TG-ND group than in the WT-ND group during the light-off period; this behavior was restored in the TG-HFD-High group. The latency of rearing during the light-off period was significantly shorter in WT-HFD group than in the WT-ND group and in the TG-HFD-High group than in the TG-ND group. The numbers of clockwise (CW) and counterclockwise (CCW) circles were significantly higher in the TG-ND group than in the WT-ND group during the light-off period; this behavior was restored in the TG-HFD-High group (Figure 6B). These results suggest that working memory dysfunction, abnormal hyperactivity, and repetitive behaviors observed in 5XFAD mice could be recovered by HFD feeding.

Thioflavin-S staining was performed to analyze the changes in amyloid plaque number in the brain, which is another pathological feature of AD. Representative images of thioflavin S-stained brain tissue in the hippocampus and hypothalamic fornix regions are shown in Figure 6C. The number of amyloid plaques in the hippocampus was significantly lower in the TG-HFD-High group than in the TG-ND group. However, no differences were found in the number of plaques in the hypothalamic fornix regions among HFD-fed groups (Figure 6C). These results indicate that the HFD might have a positive effect on the behavioral characteristics of AD, as well as on the number of amyloid plaques, an important pathological feature of AD.

## 3. Discussion

According to various clinical case analyses and meta-analysis results, the risk of AD is greater in the middle-aged high-BMI population, whereas it is lesser in the older high-BMI population [9,15,16,17,18]. Other studies suggested that the risk of AD is not only influenced by high and low BMI, but also by “lifetime changes” in BMI [19]. In a study by Yoo et al., body weight variability (defined as the body weight change) and its correlation with AD risk were evaluated in approximately 17,000 patients during 3–5 visits [20]. A high risk of AD was positively correlated with a higher weight change in the older patient group, whereas a lower weight change was associated with a low risk of AD [21]. Another study showed that middle-aged individuals with BMI > 30 and older individuals with BMI < 27 exhibited the highest risk of AD [22]. Additionally, a sharp decline in BMI in the older population was shown to increase the risk of AD [23]. However, it is difficult to understand the specific body composition contributing to these associations, as BMI is an arithmetic value based on height and weight, without accurate muscle mass, fat mass, and total body water analyses. Adipose tissue has various functions in the body, such as the storage of energy via fatty acids; production of inflammatory cytokines, chemokines, growth factors, and hormones; and storage of substances harmful to the human body [24,25]. However, adipose tissue can play both positive and negative roles in the body. Brain Aβ is transported to the periphery via LDL receptor-related peptide 1 (LRP1), glymphatic pathways, and drainage of interstitial fluid [26,27]. However, the metabolism and/or catabolism of Aβ efflux remains unclear. In in vivo histological studies, peripheral Aβ was reported to accumulate in adipose tissue, whereas in vitro studies showed that adipocytes had the potential for Aβ uptake from cultured media [28]. Although adipose tissue reduces Aβ toxicity in the body by accumulating Aβ, hypertrophy of adipose tissue mass due to excessive obesity has the potential to increase negative effects, such as inflammation.

Other clinical case studies indicated a correlation between the blood cholesterol level and risk of developing AD [29,30]. One report showed that individuals with higher levels of LDL and TAG in the blood had a higher risk of AD [31,32], while one study theorized that the elevated blood levels of LDL and TAG inhibit neuronal connections and promote the deposition of amyloid plaques in the brain [29]. Another clinical study found a negative correlation between blood HDL levels and AD risk. The study revealed that people with low blood HDL levels had a higher risk of developing AD, whereas those with high blood HDL levels exhibited a lower risk of AD development [33]. Based on these results, we speculate that HDL has a protective effect on the progression of AD.

In our study, we showed that increased adipose tissue mass and blood HDL levels had protective effects on pathological characteristics of AD such as amyloid plaque number and glial cell reactivity in the brains of HFD-fed 5XFAD female mice. It is well known that HDL not only exhibits anti-inflammatory and antioxidant effects, but it also provides structural integrity via cell membrane fluidity, guides the delivery of biomolecules (such as proteins, micro-RNA, vitamins, and hormones), and plays an important role in neurotransmitter signaling [34,35]. HDL contains cholesterol esters, TAGs, cholesterol, APO, and phospholipid spheres. APOE is the dominant type of lipoprotein in the cerebral region and is locally synthesized by astrocytes and microglia [36]. APOE2 and E3 types reduce the risk of AD, while APOE4 promotes the risk of AD by enhancing seeding and fibrillization of Aβ [37]. APOA-I is the main lipoprotein in the peripheral region and is synthesized by the liver and intestine [38]. These peripheral APOA-I HDLs can penetrate the blood–brain barrier (BBB) through the scavenger receptor class B type I (SR-BI)-mediated transcytosis system [39]. These types of HDLs play a protective role in AD progression in the brain by (1) intervening in Aβ production by binding to amyloid precursor protein and inhibiting its endocytic processes, (2) inhibiting Aβ aggregation by blocking Aβ self-assembly via hydrophobic interaction with Aβ, and (3) enhancing Aβ clearance with Aβ complexes to promote cellular uptake, degradation, and efflux of Aβ through the BBB for clearance [40]. Even though the role of peripheral APOA-I in the brain for the mitigation of AD progression has been studied, sufficient penetration of peripheral APOA-I to the brain is needed for efficient action. Additionally, APOs are lipidized by glia cells via ATP binding cassette A1 (ABCA1) activity [41]. However, unlipidated APO peptides in the extracellular matrix have the potential to initiate Aβ aggregation [42]. These observations suggest that high levels of fully functional and lipidated APO-HDLs in the cerebral and peripheral regions have protective effects on the AD brain.

In this study, we did not confirm the efficacy of HDL by directly regulating its concentration in the blood. Various methods have been used to specifically synthesize or separate HDL in the blood, such as the direct separation of HDL from serum plasma, synthesis of reconstituted HDL (rHDL) through sonication by adding APO to the lipid mixture, adding APO after the separation of lipids from blood, adding sodium cholate and APO to the lipid mixture, and synthesis of rHDL through microfluidics [40]. In future studies, we plan to examine the direct effect of HDL on AD progression by modulating the level of HDL in the blood or brain and confirm the different effects of HDL by APO type. Nonetheless, HDL and adipose tissue mass might be critical in finding novel AD therapeutic strategies against neuroinflammation and amyloid pathogenesis.

Cholesterol is essential for life and is a major structural component of cell membranes. Additionally, it is a crucial substrate for steroids, sex hormones, and adrenocortical hormones [43,44]. The formation of LDL and/or HDL is determined by the ratio of APO to cholesterol, and not by any mechanistic differences [45]. In this study, the concentration of HDL (but not LDL) in the blood was increased in HFD-fed mice. The exact mechanism of this phenomenon is unknown; however, cholesterol metabolism abnormalities might have been the cause. Increases in HDL can be caused by alterations in the de novo biosynthesis of cholesterol, release of cholesterol, and packing of HDL into plasma through ABCA1 in extrahepatic tissues [46]. Another possible mechanism is the efflux of HDL by SR-BI and its metabolism by cholesteryl esters in the liver [47]. Further research is needed to determine the reason for the increase in HDL after HFD initiation through cholesterol metabolism.

The reactivity of glial cells in the AD brain is well known [48]. Although, in our study, the reactivity of astrocytes in the hippocampus was not clearly observed in TG-ND mice, microglia were transformed to the reactive state in the hippocampal DG region in TG-ND mice. In contrast, the reactivity of astrocytes and microglia was confirmed in the hypothalamus, particularly in the fornix region. Interestingly, the reactivation of glial cells was restored by the HFD. In our TG-HFD-fed mice, the loss of working memory, repetitive behavior, and hyperactivity during the light-off period were also partially recovered by the HFD. The number of amyloid plaques was decreased in the hippocampal area of the HFD group. Another brain function of APOs is their anti-inflammatory effect. Several studies have confirmed that APOA-I and APOE function as immunosuppressants in various brain diseases, although the exact molecular mechanisms have not been elucidated [49]. Activated glial cells decrease the degradation and clearance of Aβ [50]. The increase in the cerebral inflammatory response in AD not only accelerates the accumulation of amyloid plaques, but also causes neuronal damage in the brain [36]. In our study, the reactivity of glial cells was decreased in the brain, with increased peripheral levels of HDL and APOA-I, under the HFD, which could diminish the inflammatory response of the brain and restore amyloid clearance.

Interestingly, after HFD initiation, female TG-HFD mice could be clearly divided into two groups (normal and obese groups) based on the body weight change. Initially, we assumed that this was caused by differences in food intake due to grouped housing. However, the use of individual housing confirmed that the body weight changes were not due to a difference in food and/or calorie intake. EE was lower in the TG-HFD-High group than in the TG-ND group, while it was higher in the TG-ND group than in the WT-ND group, during the light-off period. However, no significant difference in EE was found between the TG-HFD-High and TG-HFD-Low groups. Therefore, we examined agouti-related peptide (AgRP) neurons and pro-opiomelanocortin (POMC) neurons in the arc region, which, along with leptin and ghrelin, play an important role in food intake and energy metabolism [51,52]. The reactivity of astrocytes and microglia in the arc region was not different between TG-HFD-high and TG-HFD-Low groups. Furthermore, amyloid plaque accumulation was not observed in most regions of the hypothalamus, particularly in the arc region, in the TG groups. Therefore, the difference in body weight changes between TG-HFD-High and TG-HFD-Low groups may be due to other unknown mechanisms associated with individual hormonal differences. Future studies should analyze the changes in leptin and ghrelin levels, as well as the cell-type specific changes in AgRP and POMC neurons, between the two groups.

Moreover, after HFD initiation, the male HFD mice could also be divided into two groups, but the difference in relative body weight changes between the TG-HFD-High and TG-HFD-Low was not as obvious as that with female mice, and the TG-HFD-Low group also showed a significant increase in body weight compared to that in the TG-ND group (unlike the female TG-HFD-Low mice). Additionally, the grouping based on the body weight within the HFD group was not clearly established in male mice. For these reasons, in this study, female mice were used as the main experimental group although the trend of most of the data in male mice was comparable with female mice. The analysis of fat weight, blood cholesterol, glucose tolerance, the reactivity of glia cells, the number of amyloid plaques and behavior in male mice was shown in Supplementary Figures S1–S3.

The maintenance and formation of adipose tissue are extremely essential to the body [53]. Adipose tissue is a complex organ composed of adipose stem cells, adipocytes, and various other cell types. These novel adipocytes are continually generated in adult humans and rodents [54,55]. However, the mechanism of adipose tissue formation remains largely unknown. In our study, although excessive energy was accumulated in the body under the HFD, lipid droplet accumulation did not occur in the adipose tissue in the SCF and VF of the TG-HFD-Low group. Further studies are needed to elucidate the cause of the differences in adipose tissue amount between TG-HFD-High and TG-HFD-Low groups by analyzing the number of adipose stem cells in the adipose tissue and the concentration of various hormones that promote the differentiation of adipose stem cells.

The mouse is a nocturnal animal, and there is a possibility that the results of a behavioral experiment are more robust during the light-off period [56]. Interestingly, in our LABORAS data, some groups showed significantly different behavior in only the light-off period; both female and male TG-ND groups exhibited increased total distance and circling behavior in the light-off period (Figure 6, Supplementary Figure S3). Other studies have also shown hyperactivity on behavioral analysis in AD mice models during the light-off period. Mislocation of aquaporin-4, hyperphosphorylation of tau, and altered circadian rhythms have been suggested as the causes of this hyperactivity [56,57].

In conclusion, 5XFAD mice fed a HFD showed a decrease in amyloid plaques in the hippocampus, reduction in glial activation in the hypothalamic fornix, and enhancement of behavioral abnormalities, along with a marked increase in blood HDL and APOA-I, but not LDL. These results suggest that adipose tissue mass and peripheral HDL might be crucial factors in the treatment of AD. However, the exact molecular mechanisms require further investigation.

## 4. Materials and Methods

### 4.1. Animals Experiments and Sampling

5XFAD AD model TG mice were purchased from Jackson Laboratories (strain: B6J-TG [APP Sw, Fl, Lon, PS1, M146L, L286V] 6799Vas/J). The TG mice were maintained by crossing hemizygous TG mice with C57B6J mice. Two-month-old male and female mice were used for the experiments. Before HFD feeding, three to five mice were housed per cage with free access to food and water, a 12 h:12 h light-dark cycle, and 55 ± 5% humidity at 25 ± 1 °C. To induce the obese model, one mouse was housed per cage with a 60% fat diet for the HFD group, while one mouse was housed per cage with a 10% chow diet for the ND group. The diet was changed every week, and the body weight and consumed food weight were measured weekly. After conducting all behavioral experiments, 2,2,2-tribromoethanol (150 mg/kg, Avertin, Sigma, St. Louis, MO, USA) was injected intraperitoneally into the mice to induce anesthesia. The mice were sacrificed by serial perfusion with 1× phosphate-buffered saline (PBS, pH 7.2) for 5 min and fixed in 4% paraformaldehyde (PFA). Fixed brain and fat tissues, such as SCF, VF, and BF were collected from the mice.

### 4.2. Metabolic Tests

#### 4.2.1. Indirect Calorimetry

The mice were fasted from 7:00 p.m. to 11:00 a.m. for IC. After measuring the body weight, mice were placed in the bedded home cage of the OxyletPro system (Panlab, Barcelona, Spain) with food and water. Each group of mice was fed their respective diet with water, i.e., 60% fat diet for the HFD group and 10% normal chow diet for the ND group. The system measured respiratory metabolism via vO_2_ and vCO_2_ from 3:00 p.m. to 11:00 a.m. The mice were analyzed every 30 min for vO_2_/vCO_2_ changes, RQ, and EE.

#### 4.2.2. Glucose Tolerance Test

The mice were fasted from 7:00 p.m. to 11:00 a.m. for GTT. After measuring the body weight of the mice, the tail was punctured with an adjustable tip for measuring the baseline blood glucose levels via a glucometer strip and reader (#AgaMatrix Presto, AgaMatrix, NH, USA). Next, the mice were administered a 10% (*w*/*v*) glucose solution in a 10 mL/kg volume using a 1-mL insulin syringe. The change in blood glucose level was then measured at 15, 30, 60, and 90 min after glucose injection.

### 4.3. Behavior Tests

#### 4.3.1. Y-Maze Test

The Y-maze test was performed as previously described [58]. The Y-maze comprised three equal arms (35 × 15 × 9 cm) made of a gray acrylic plate. The mice were kept in the test room for 1 h for habituation. The Y-maze was cleaned with 30% ethanol (EtOH). After habituation, the mice were placed in the center of the Y-maze. The mice were allowed to freely explore the maze for 5 min. Video recording was obtained using SMART video tracking software version 3.0 (Panlab, Barcelona, Spain). The alternation ratio of each arm and the number of arm entries were recorded.

#### 4.3.2. The Laboratory Animal Behavior Observation, Registration and Analysis System Test

After measuring the body weight of the mice, mice were placed with food and water in the bedded home cage of a LABORAS (Metris, Hoofddorp, The Netherlands). The LABORAS test was performed from 3:00 p.m. to 11:00 a.m. During the test period, a 60% fat diet was provided to the HFD group, and a 10% normal chow diet was given to the ND group, along with water. The system automatically measured the latency and frequency of various basic mouse behaviors, such as locomotion, immobility, distance, climbing, rearing, grooming, CW circling, and CCW circling, by tracking the mice movement. These behaviors were analyzed every 1 h.

### 4.4. Histological Procedures

#### 4.4.1. Tissue Slicing

Tissue slicing was performed using a protocol previously described [59]. Brains were fixed with 4% PFA for 24 h. The fixed brains were then transferred into a 30% sucrose solution for 48 h. Brain tissue was sectioned into 30-μm thick slices using a cryotome. The fat tissues, including SCF, VF, and BF, were fixed in 10% neutral buffered formalin. After paraffin embedding, the fat tissues were sectioned into 5-μm thick slices on glass slides using a microtome.

#### 4.4.2. Immunofluorescence Staining

Immunofluorescence staining was performed as previously described [60,61]. Brain sections were washed three times with PBS and permeabilized with ice-cold methanol at −20 °C for 10 min. The brain sections were again washed three times with PBS and heated in a 95 °C water bath for 30 min with 10 mM citrate acid (pH 6.0) for antigen retrieval. The sections were then blocked with 2% bovine serum albumin and 0.3% Triton X-100 in PBS for 1 h. Primary antibodies, GFAP (rabbit, 1:1000, Z0334, DAKO, Santa Clara, CA, USA) and Iba1 (mouse, 1:1000, MABN92, Millipore, Darmstadt, Germany), were applied overnight at 4 °C. Next, secondary antibodies, anti-rabbit-488 (Donkey, 1:200, A21206, Thermo Fisher, Waltham, MA, USA) and anti-mouse-555 (Donkey, 1:200, A31570, Thermo Fisher, Waltham, MA, USA), were applied for 2 h at 24 °C. Finally, the brain sections were counterstained with To-pro (1:1000, T3605, Thermo Fisher, Waltham, MA, USA) for nuclear staining and mounted for observation. The stained brain tissue samples were imaged using confocal microscopy (Confocal-A1R-MP, Nikon Corporation, Tokyo, Japan).

#### 4.4.3. Hematoxylin and Eosin and Thioflavin-S Staining

For general morphology and fat lipid analyses, SCF, VF, and BF were stained with H&E. Briefly, the fat tissues on glass slides were deparaffinized by serial processing. The specimens were then incubated two times with xylene for 2 min, two times with 100% EtOH for 2 min, and finally with 95% EtOH for 2 min. The deparaffinized samples were stained with hematoxylin for 3 min and eosin for 45 s. The stained fat tissues were washed once with 95% EtOH for 1 min, two times with 100% EtOH for 1 min, and two times with xylene for 2 min, followed by mounting for observation. The stained fat tissue samples were imaged using a Panoramic Slide Scan (3dHistech, Budapest, Hungary), and the size of the lipid droplets was analyzed using ImageJ and FIJI (NIH, Bethesda, MD, USA).

For amyloid plaque analysis, brain tissues were stained with thioflavin S. The thioflavin-S staining protocol has been described previously [61]. Briefly, brain sections were washed three times with PBS. The specimens were then incubated with 1% thioflavin S solution for 8 min at room temperature. The stained samples were washed with 85% EtOH two times for 5 min and 95% EtOH for 5 min. Finally, the brain sections were washed three times with PBS and mounted for observation. The stained brain tissue samples were imaged via a Panoramic Slide Scan (3dHistech, Budapest, Hungary), and the number of amyloid plaques was analyzed using a Stereo Investigator (MBF Bioscience, Williston, VT, USA).

### 4.5. Serum Cholesterol Analysis

The protocol for the analysis of serum APOA-I levels has been described in previous studies [62,63]. Briefly, blood was collected from the infraorbital plexus using microhematocrit capillary tubes (2901000, Marienfeld, Baden-Württemberg, Germany). After 1 h incubation at room temperature, the serum was separated from the supernatant by centrifugation. The serum samples were stored at −80 °C for further analyses. APOA-I levels in the mouse serum samples were measured according to the manufacturer’s instructions (ab238260, Abcam, Cambridge, UK). All serum samples were diluted to 1:20,000 in a sample dilution buffer. Each sample was measured at an absorbance of 450 nm in duplicate. TAG, CHO, LDL, and HDL levels were measured according to the manufacturer’s instructions (L-Type HDL-C M and L-Type LDL-C M, Wako, Osaka, Japan). All serum samples were diluted to 1:10 in PBS for analysis. Each sample was measured at an absorbance of 600 nm in duplicate.

### 4.6. Statistical Analysis

Data are expressed as the mean ± standard error of the mean. One-way analysis of variance followed by Tukey’s HSD post hoc analysis was performed to determine statistical significance using PRISM 9 (San Diego, CA, USA). Statistical significance was set at *p* < 0.05.

## Data Availability

The data that support the findings of this study are available from the corresponding author upon reasonable request.

## Supplementary Materials

The following supporting information can be downloaded at: https://www.mdpi.com/article/10.3390/ijms232012296/s1.
