# Supplementary material for: Effect of Obesity and High-Density Lipoprotein Concentration on the Pathological Characteristics of Alzheimer’s Disease in High-Fat Diet-Fed Mice
Source: Int J Mol Sci. 2022 Oct 14;23(20):12296. doi: 10.3390/ijms232012296 (PMC9603479; doi:10.3390/ijms232012296)
Supplement: Supplementary file 1 [file ijms-23-12296-s001.zip › ijms-1884574 - supplementary.pdf]

International Journal of
Molecular Sciences

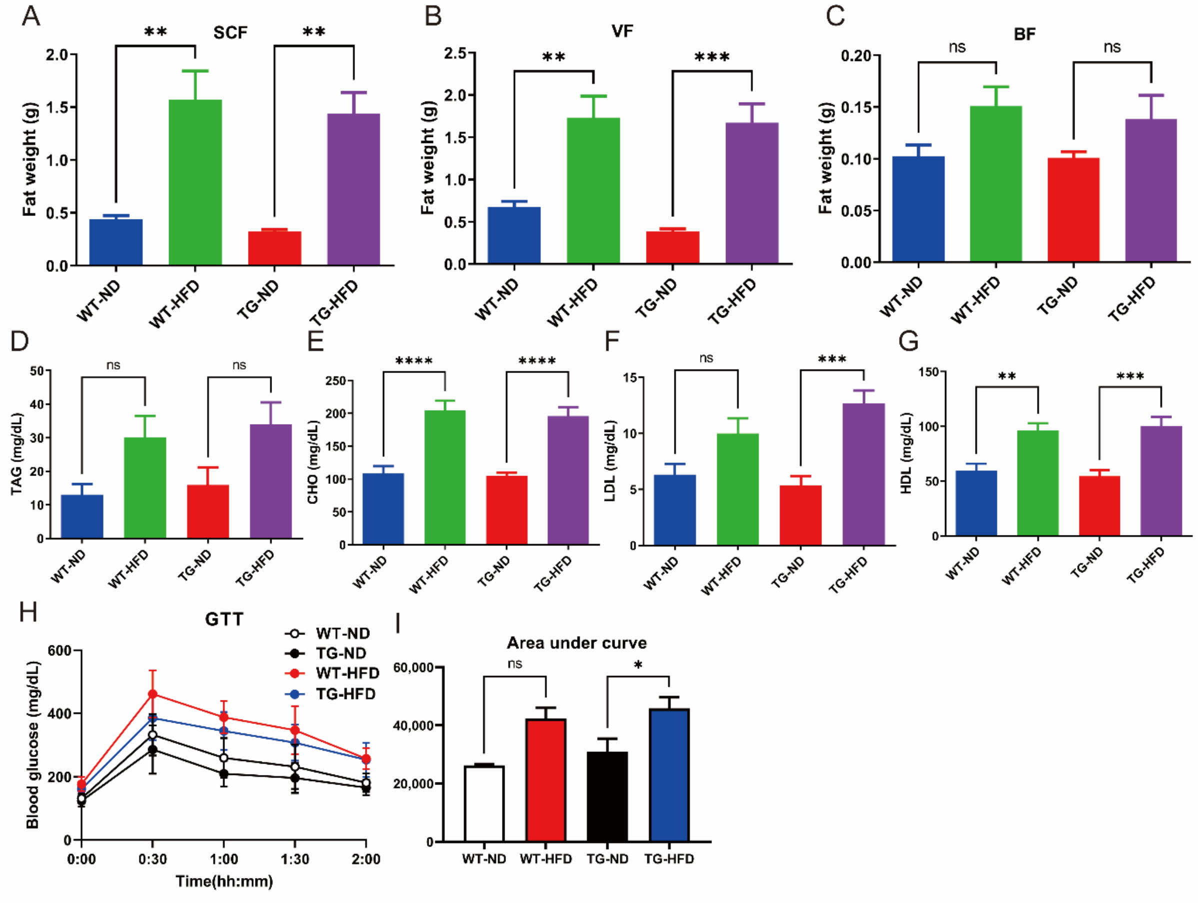

Supplementary Figure S1. Analysis of the fat weight (**A**–**C**), the blood cholesterol (**D**–**G**), and the glucose tolerance test (GTT) (**H**,**I**) changes under a high-fat diet (HFD) in the male 5XFAD Alzheimer's disease transgenic mouse model. ns (not significant), *$p < 0.05$, **$p < 0.01$, ***$p < 0.001$, and ****$p < 0.0001$ (one-way analysis of variance with Tukey post hoc analysis). The number of animals in each group ranged 3–6. Data are presented as the mean ± standard error of the mean. ND, normal chow; WT, wildtype; TG, transgenic.

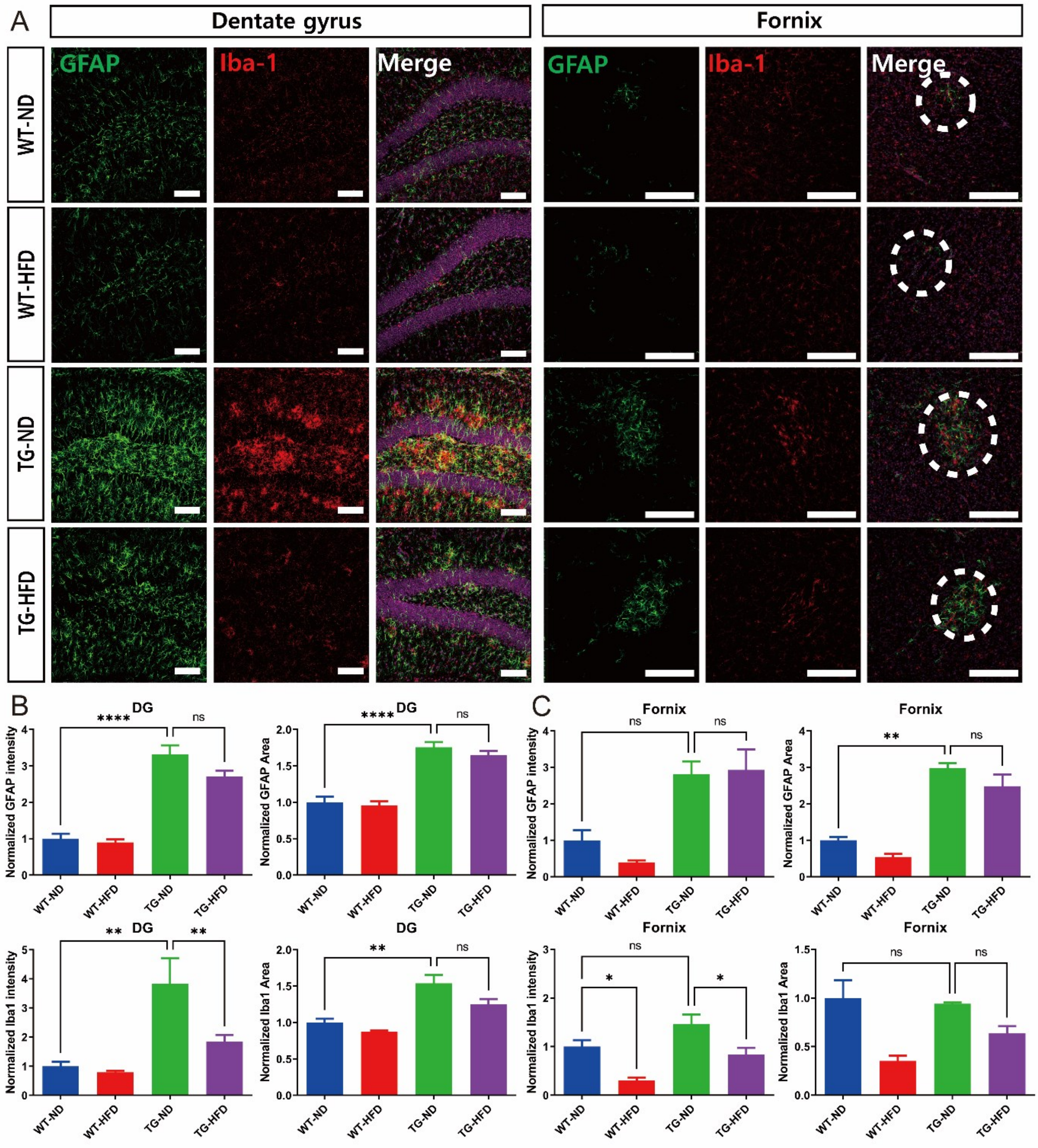

Supplementary Figure S2. Analysis of astrocyte and microglia reactivity in the hippocampal dentate gyrus (DG) (**A**,**B**) and the hypothalamic fornix region (**A**,**C**) under a high-fat diet (HFD). Representative immunofluorescence images of glial fibrillary acidic protein (GFAP) and ionized calcium binding adaptor molecule 1 (Iba1) staining in the male 5XFAD Alzheimer's disease transgenic mouse model. Scale bars = 100 μm, ns (not significant), *$p < 0.05$, **$p < 0.01$, and ****$p < 0.0001$ (one-way analysis of variance with Tukey post hoc analysis). The number of animals in each group ranged 3–8. Data are presented as the mean ± standard error of the mean. ND, normal chow; WT, wildtype; TG, transgenic.

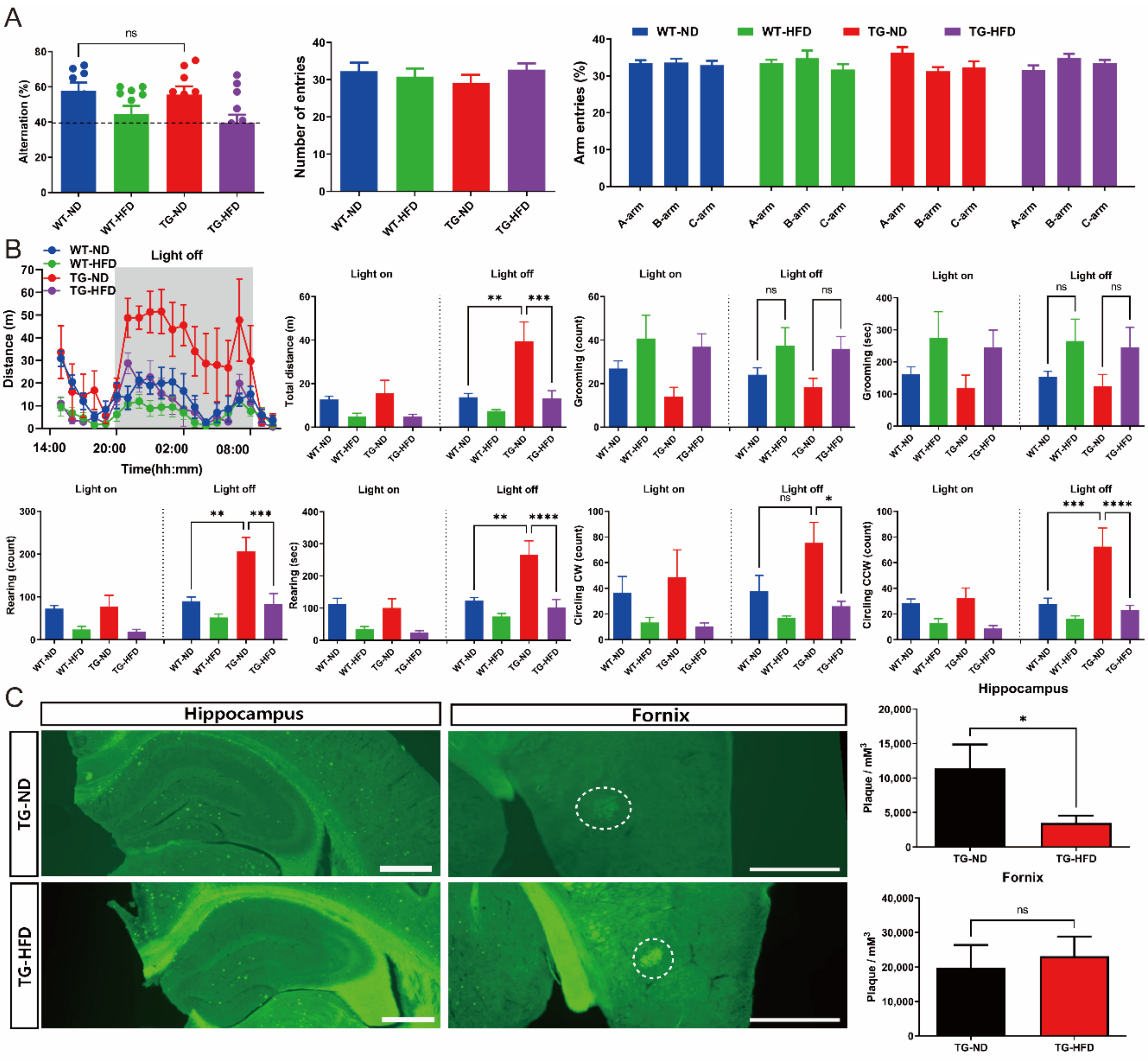

Supplementary Figure S3. Investigation of behavioral changes and amyloid plaque number under a high-fat diet (HFD) in the male 5XFAD Alzheimer's disease transgenic mouse model. Bar graphs of spontaneous alternations in the Y-maze (**A**, left panel), number of entries (**A**, center panel), and percent of arm entries (**A**, right panel). Analysis of the general behavior of mice using the Laboratory Animal Behavior Observation, Registration and Analysis System (LABORAS) test. Total distance, duration, and frequency of grooming, rearing, and clockwise and counterclockwise circling in light-on and -off conditions (**B**). Representative images of thioflavin-S staining in the hippocampal and fornix regions (**C**, left panel), bar graph of the amyloid plaque density in the hippocampal and fornix regions (**C**, right panel). Scale bars = 500 µm, ns (not significant), *$p < 0.05$, **$p < 0.01$, ***$p < 0.001$, and ****$p < 0.0001$ (one-way analysis of variance with Tukey post hoc analysis). The number of animals in each group ranged 3–6. Data are presented as the mean ± standard error of the mean. ND, normal chow; WT, wildtype; TG, transgenic.
